# Supplementary material for: Purcell-enhanced quantum yield from carbon nanotube excitons coupled to plasmonic nanocavities
Source: Nat Commun. 2017 Nov 10;8:1413. doi: 10.1038/s41467-017-01777-w (PMC5680202; doi:10.1038/s41467-017-01777-w)
Supplement: Supplementary file 1 — Supplementary information [file 41467_2017_1777_MOESM1_ESM.pdf]

Supplementary Note 1. Numerical simulations of plasmonic nanocavities

To guide the structural design of the plasmonic nanoantenna before nanofabrication we have carried out finite-difference time-domain (FDTD) simulations using Lumerical FDTD Solutions software package[1] to determine the geometry parameters that allow plasmon resonance energy matching with the known SWCNT emission energy, the expected Purcell enhancement, the light collection efficiency, and the local heat map. A total-field scatter-field source was used to excite the plasmonic structures and a simulation region with 1 nm uniform mesh size in *x*-, *y*-, and *z*-directions was defined. The dielectric constants data of gold measured by Johnson and Christy[2] were used to model the plasmon resonances. The dielectric function for the glass substrate was modeled from the measurement of Palik[3]. Frequency-domain field and power monitors were placed near the sample surface to record the near-field electrical field intensity. A built-in analysis module was placed to calculate the mode volume. To determine the plasmon resonance wavelength we varied the lateral extent of the triangle, layer thickness, and gap size. The lateral extent was found to have the largest influence on wavelength with a linear dependence on triangle side length, while gap size and layer thickness have only minor influence and thus have been kept optimized to maximally enhance the field intensity of the plasmonic gap mode. Given that the center of the plasmon mode should be around 870 nm to support optical emission from (5,4), (6,4) and (9,1)-chirality SWCNTs we estimated that the optimized side length of the bowtie structure should be 250 nm, as shown in **Supplementary Fig. 1a**. The corresponding field enhancement profile was acquired by a power monitor resulting in the spatial field distribution in the *x*-*z* plane that highlights the electric field above the nanocavity where the SWCNT is placed (**Supplementary Fig. 1b)**.

To calculate the theoretical Purcell factor, $F_\mathrm{p} = \frac{3}{4\pi^2}\left(\frac{\lambda}{n}\right)^3 \frac{Q_\mathrm{eff}}{V}$, where λ, *n*, Q_eff, and *V* are the cavity wavelength, refractive index, effective quality factor, and mode volume, we have simulated the plasmonic mode profile and calculated the mode volume. The effective mode volume of an emitter located at the mode antinode is given by the integral V_{eff}= $\int\epsilon|(\boldsymbol{r})|^2\mathrm{d}x/\max(\epsilon|\boldsymbol{E}(\boldsymbol{r})|^2)$. We note that this definition of mode volume describes dielectric nanocavities well but was found to underestimate the resulting F_P by up to one order of magnitude for isolated metal nanoparticles since a set of normal modes can generally not be defined for dispersive absorbing media[4]. However, the discrepancy of the mode volume calculation is less significant in the dimer plasmonic system featuring gap-modes when nanoparticles are strongly coupled to each other forming hybridized plasmonic modes. In this case the discrepancy at a distance of 2 nm is only a correction of 50%, which is a rate enhancement of 800 using the classical formula versus 1200 with the Green's function approach in Figure 2 of Ref.4. Since the purpose of the theoretical Purcell factor is to provide an order of magnitude estimate to guide the design of the plasmonic chip the 50% error by using the classical mode volume calculation is justified here.

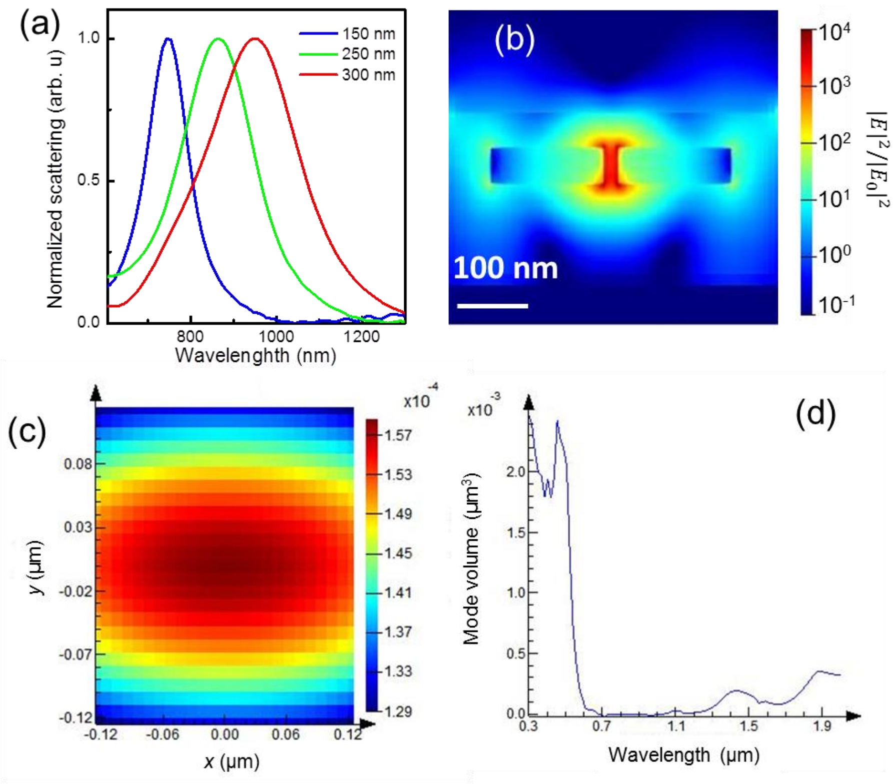

Supplementary Figure 1 FDTD simulation results of plasmonic gold nanocavities with bowtie shape. **a**, Finite-difference time-domain (FDTD) method simulated scattering spectrum peaking at 870 nm at a Q factor of 6 (green solid line). The length in the legend corresponds to the bowtie side length used for each simulated spectrum. **b,** Intensity enhancement $|E|^2/|E_0|^2$ shown in the *x*-*z* plane to highlight the field above the nanocavity where the SWCNT is placed. (Scale bar shows *x* direction scaling while *z* direction is extended.) **c,** Mode plot at localized surface plasmon resonance. **d,** Mode volume as a function of probe wavelength showing ultra-small mode volume in the 900 nm band.

The resulting mode profile is shown in **Supplementary Fig. 1c** while the dependence of mode volume on probe wavelength is shown in **Supplementary Fig. 1d**. The effective quality factor Q_{eff} of a coupled emitter-mode system can be obtained by $\frac{1}{Q_{\mathrm{eff}}} = \frac{1}{Q_{\mathrm{cav}}} + \frac{1}{Q_{\mathrm{x}}}$, where Q_{cav} and Q_x are the quality factors of cavity mode and exciton emission, respectively. From the white light source scattering spectrum one can calculate Q_{cav} from the full width half maximum (FWHM), that is $Q_{\mathrm{cav}} = \frac{\omega_c}{\mathrm{FWHM}}$, where ω_c is the resonance frequency. Since the FWHM of the zero-phonon line (ZPL) from the carbon nanotube exciton emission is 18 μeV to 10 meV, that is several orders of magnitude narrower than the cavity mode, we can assume $Q_{eff} \approx Q_{cav} = 6$. As stated in the main text, we further added a 2 nm Al_2O_3 spacer layer to maximize the radiative

recombination rate over the NR rate and to prevent PL quenching, spectral diffusion, and electric shortening of the gap mode. This will cause the optical mode to explore the additional dielectric medium and thereby cause the mode volume to be larger as compared to a bare nanocavity. Using a 2 nm dielectric spacer the mode volume was found to be V_{eff} = 44000 nm^3 and the resulting Purcell factor is F_p= 6591.

In addition, the collection efficiency of coupled and reference SWCNTs was determined by FDTD simulations resulting in far-field projection pattern shown in **Supplementary Fig. 2a**. For coupled SWCNTs simulation, a dipole source has been placed on the structure shown in **Fig.1a**. The far-field pattern is generated using a frequency-domain field power monitor. The objective lens has a numerical aperture NA = 0.81 leading to ±54 degree collection angle. To simulate the reference SWCNT, we removed the plasmonic cavity and placed the dipole source on the same planar metal-mirror substrate with 60 nm SiO_2 spacer and 2 nm Al_2O_3. This structure corresponds to the case where the reference SWCNTs are located. All results are calibrated with the dipole source power to extract the collection efficiency. The calculated collection efficiency for the plasmonic cavity was found to be η_c = 64%, while without the cavity the planar gold mirror substrate yields η_{c0} = 32%, and dipoles located on the bare substrate yield 13%. The additional enhanced light extraction compared to the reference SWCNTs on the metal mirror is at most ε = 2. We also calculate the collection efficiency as a function of displacement between the emitter and the field maximum as shown in **Supplementary Fig. 2b**. The far-field light collection efficiency slowly decreases to 1.4 times even at 80 nm away from the field maximum. From this study, it is clear that the collection efficiency enhancement provided by the plasmonic nanocavity, albeit omnipresent, contributes the smallest factor (≤ 2) to the total light enhancement from the SWCNTs that we measure in **Fig. 4a** of the main manuscript.

To estimate the metal loss of our plasmonic cavities, we simulated a cavity containing an electric dipole oscillating along the long axis of the bowtie structure and positioned in the gap region. We set the emission wavelength of the dipole emitter ranging from 850 nm to 950 nm to cover the emission wavelength of SWCNTs with different chirality. This dipole is assumed with a 100% radiative emission rate in order to simulate the cavity loss independent of the emitter. We estimated the power emitted out of the cavity and normalized it to the total power radiated by the dipole emitter. In this way, we can estimate the metal loss since the internal quantum yield of the dipole emitter is unity. As shown in **Supplementary Fig. 2c**, approximately 71-78% of the power in the plasmon mode is emitted radiatively, as measured by a near-field power monitor in the FDTD simulation. As a result, the metal loss amounts to 22-29%, depending on wavelength. This simulation result is in a good agreement with metal loss values of 25% reported by Russel *et al*. who utilized comparable plasmonic gap-mode cavities[5].

Furthermore, we carried out heat profile simulations and estimated the temperature raise upon laser irradiation using Lumerical Solutions finite-element DEVICE package. To this end, the absorption efficiency of the gold nanocavity for 780 nm laser irradiation was first found via FDTD simulations. The total power of the heat source was corrected by the absorption and placed to be of the same size as the Abbe-limited laser spot Airy-disc size. The heat simulation module defined in the simulation region contains a temperature monitor to read the temperature difference. The thermal boundary condition is set to 4 K at $-z$ direction as the base temperature. By sweeping the incident power from 0.01 mW to 1 mW, one can calculate the temperature raise ΔT as a function of excitation power. The resulting temperature raise as well as the heat distribution map on the metal surface are shown in **Fig. 5d** of the main text.

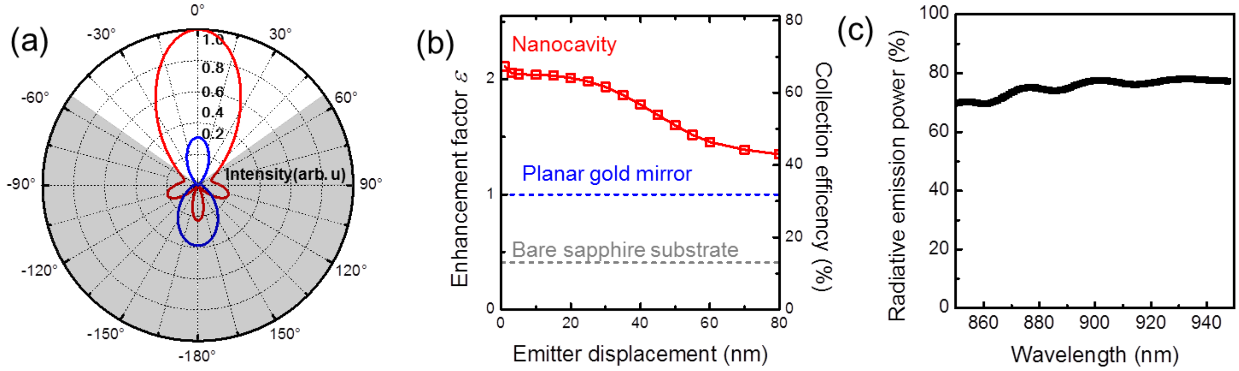

Supplementary Figure 2 Light collection efficiency calculation. **a**, FDTD calculated far-field projection pattern of an reference carbon nanotube (blue line) and coupled carbon nanotube (red line). **b**, Collection efficiency enhancement ε as a function of the displacement of emitter away from the field maximum. The red squares show the light collection efficiency at various emitter displacements from the nanocavity center while the blue dashed line illustrates the collection efficiency without the nanocavity but on planar gold mirror with 60 nm SiO_2 and 2 nm Al_2O_3, where the reference SWCNTs are located. Note that ε is normalized to the collection efficiency of the planar gold/dielectric substrate where the reference SWCNTs are located. The gray dashed line shows comparison to the bare sapphire substrate which yields ε=5 at zero displacement. All data are calculated for 860 nm, that is (5,4) SWCNTs. **c.** The amount of power emitted radiatively from the plasmon mode as a function of emitting wavelength.

Supplementary Note 2. Three-level rate equation analysis

It is well known that excitons in SWCNTs are strongly spatially localized at cryogenic temperatures, as is evident from the observation of photon antibunching in **Fig.3c** of the main text. In this case the exciton emission under continuous laser excitation can be described as an effective three-level system. To describe the PL saturation behavior observed in **Fig. 4a** of the main text we followed Hofmann *et al* [6] and applied a standard coupled rate equation analysis to quantitatively describe the power dependent response, were the absolute ground state is denoted by $|G\rangle$, the exciton ground state by $|X\rangle$ and the excited exciton state by

$|X^*\rangle$. In this case the evolution of population with time is given by the set of coupled rate equations:

$$\dot{\rho_G} = -\rho_G\gamma_{\mathrm{abs}} + \rho_X\gamma_{\mathrm{rad}} \quad (1)$$

$$\dot{\rho_{X^*}} = \rho_G\gamma_{\mathrm{abs}} - \rho_{X^*}\gamma_{\mathrm{rel}} \quad (.2)$$

$$\dot{\rho_X} = \rho_{X^*}\gamma_{\mathrm{rel}} - \rho_X\gamma_{\mathrm{rad}}, \quad (3)$$

where the total state population $\rho_G + \rho_X + \rho_{X^*}$ is normalized to 1, and $\gamma_{\mathrm{abs}}, \gamma_{\mathrm{rad}}, \gamma_{\mathrm{rel}}$ are the absorption, radiative recombination, and relaxation rates respectively. We assume that the relaxation rate from excited exciton state to the exciton ground state is much faster than all other rates and can be neglected. For this case the steady-state, solution is given by:

$$\rho_X = \frac{\gamma_{\mathrm{abs}}}{\gamma_{\mathrm{abs}}+\gamma_{\mathrm{rad}}} \quad (.4)$$

The PL intensity is proportional the product of ρ_X and γ_{rad} and the detection efficiency. The latter can be eliminated by normalizing to the saturation intensity I_0 of the PL emission which yields:

$$I = I_0\frac{\gamma_{\mathrm{abs}}}{\gamma_{\mathrm{abs}}+\gamma_{\mathrm{rad}}} \quad (5)$$

Since γ_{abs} is linearly proportional to the incident laser intensity I_{laser} one can write $I_{\mathrm{laser}} = \alpha\gamma_{\mathrm{abs}}$ and rewrite **Supplementary Equation 5** as follows:

$$I = I_0\frac{I_{\mathrm{laser}}}{I_{\mathrm{laser}}+\alpha\gamma_{\mathrm{rad}}} \quad (6)$$

Best fits were found by taking the saturation intensity from the pump intensity dependent experiment shown in **Supplementary Fig. 3**, and the radiative rates from the time-correlated single photon counting (TCSPC) experiment in **Fig. 4g**, and varying the only free parameter α to fit the data.

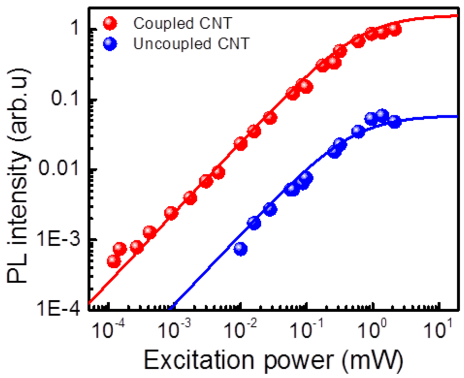

Supplementary Figure 3 Three-level rate equation analysis of E_{11} exciton emission. Photoluminescence intensities of the E_{11} zero-phonon line (ZPL) as a function of excitation power. Red circles are data for a coupled SWCNT and blue circles are from a reference SWCNT on the metal mirror with dielectric. Solid lines are fits to a three-level rate equation model. The onset of saturation at highest pump powers is apparent.

Supplementary Note 3. Derivation of Purcell factor and quantum yield of SWCNT coupled to plasmonic nanocavities in the presence of metal losses

The total decay rate through each decay channel for reference SWCNTs without coupling to plasmonic nanocavities is:

$$\gamma_{\mathrm{off}} = \gamma_{\mathrm{R}} + \gamma_{\mathrm{NR}} \tag{7}$$

where $\gamma_{\mathrm{R}}$and γ_{NR} are the radiative and nonradiative decay rates. The coupling to plasmonic nanocavities enhances the radiative rate by the Purcell effect and induces a new decay channel through metal loss with rate γ_{M}. Therefore, the total decay rate γ_{on} of the coupled SWCNTs can be expressed as:

$$\gamma_{\mathrm{on}} = (F_{\mathrm{p}} + 1)\gamma_{\mathrm{R}} + \gamma_{\mathrm{NR}} + \gamma_{\mathrm{M}} \tag{8}$$

where F_p is the Purcell factor that enhances the radiative rate. The ratio of these two rates is given by

$$\frac{\gamma_{\mathrm{on}}}{\gamma_{\mathrm{off}}} = \frac{(F_{\mathrm{p}}+1)\gamma_{\mathrm{R}}+\gamma_{\mathrm{NR}}+\gamma_{\mathrm{M}}}{\gamma_{\mathrm{R}}+\gamma_{\mathrm{NR}}} \tag{9}$$

which was determined by the time-resolved measurements (**Fig. 4**). For quantum emitters like quantum dots with near 100% internal quantum yield of fluorescence η (emission quantum yield) the nonradiative rate γ_{NR} is much smaller than the radiative rate γ_{R} and the Purcell enhancement provided by the cavity mode goes directly into the measured rate enhancement in an experiment. This is however not the case for quantum emitters like SWCNTs which suffer from low internal quantum yields of typically 2%. In this case most of the Purcell enhancement of γ_{R} is used to catch up with γ_{NR}. Even at an order of magnitude enhancement of γ_{R} the total measured rate γ_{on} increases only by a few percent, while F_p=50 would just double the measured rate γ_{on}.

In order to determine the underlying Purcell factor from the measured rates we note that quantum yield η is defined by the ratio of the number of photons emitted to the number of photons absorbed[7]:

$$\eta_{\mathrm{off}} = \frac{\gamma_{\mathrm{R}}}{\gamma_{\mathrm{R}}+\gamma_{\mathrm{NR}}} \tag{10}$$

$$\eta_{\mathrm{on}} = \frac{(F\mathrm{p}+1)\gamma_{\mathrm{R}}}{(Fp+1)\gamma_{\mathrm{R}}+\gamma_{\mathrm{NR}}+\gamma_{\mathrm{M}}} \tag{11}$$

The Purcell enhanced emitter has thus also a cavity-enhanced quantum yield η_{on} that can be significantly larger compared to the reference system, since the Purcell enhanced radiative rate catches up with all nonradiative losses in the system. Ultimately η_{on} cannot reach unity in plasmonic systems since the metal loss cannot be entirely avoided. At best, for high enough Purcell factors, the cavity-enhanced quantum yield saturates at values determined by the metal loss rate γ_{M}. Metal loss is the dominant loss in planar metal plasmonics but was shown to play only a minor role in nanogap plasmons. For example, at dielectric spacer distance of 2 nm comparable to our system about 75% of the power of the gap plasmon is emitted radiatively while 25% of the power is lost in the metal dissipation[5].

In order to determine the Purcell factor and quantum yield in the presence of metal loss we note that the difference in total rates measured on and off the nanocavity eliminates the nonradiative rate of the quantum emitter:

$$\gamma_{\mathrm{on}} - \gamma_{\mathrm{off}} = F_{\mathrm{p}}\gamma_{\mathrm{R}} + \gamma_{\mathrm{M}}\,, \tag{12}$$

which is the total rate enhancement including metal loss. Here we assume that γ_{NR} is a property of the SWCNT that is solely determined by the dark-state trapping rate responsible for low exciton quantum yields and thus does not change upon coupling to the nanocavity. The additional plasmonic metal loss is described by γ_{M}. The fraction of radiative rate enhancement over the total rate enhancement is then given by:

$$\frac{F_{\mathrm{p}}\gamma_{\mathrm{R}}}{F_{\mathrm{p}}\gamma_{\mathrm{R}}+\gamma_{\mathrm{M}}} \tag{13}$$

This branching between radiative emission and additional metal loss is well-known from previous experiments and theory to strongly depend on the dielectric spacer distance[5]. Assuming 2 nm dielectric spacer like in our work one finds a ratio of 75% radiative to 25% metal loss corresponding to

$$\frac{F_{\mathrm{p}}\gamma_{\mathrm{R}}}{F_{\mathrm{p}}\gamma_{\mathrm{R}}+\gamma_{\mathrm{M}}} = 0.75 \text{ , and } \frac{\gamma_{\mathrm{M}}}{F_{p}\gamma_{\mathrm{R}}+\gamma_{\mathrm{M}}} = 0.25 \tag{14}$$

Inserting (14) into (9) one can extract the metal-loss corrected Purcell factor Fp given by:

$$F_{\mathrm{p}} = 0.75\left(\frac{\gamma_{\mathrm{on}}}{\gamma_{\mathrm{off}}} - 1\right)\,{\eta_{\mathrm{off}}}^{-1} \tag{15}$$

Accordingly, the cavity-enhanced quantum yield of the coupled system can be calculated from

$$\eta_{\mathrm{on}} = \frac{(F\mathrm{p}+1)\,\gamma_{\mathrm{R}}}{\gamma_{\mathrm{on}}} = \frac{(F\mathrm{p}+1)\,\gamma_{\mathrm{off}}\,\eta_{\mathrm{off}}}{\gamma_{\mathrm{on}}} = \frac{(F_{\mathrm{p}}+1)\,\eta_{\mathrm{off}}}{\frac{\gamma_{\mathrm{on}}}{\gamma_{\mathrm{off}}}} \tag{16}$$

Supplementary Equations 15 and **16** have been used to calculate the Purcell factors and cavity enhancement quantum yields from the measured rate enhancement $\frac{\gamma_{\mathrm{on}}}{\gamma_{\mathrm{off}}}$ values as well as the known quantum yield η_{off} of the reference PFO-BPy-wrapped SWCNTs, as shown in **Fig. 4j** of the main manuscript.

Parameter	Definition
γ_{off}	total decay rate of reference SWCNTs on planar gold mirror
γ_{on}	total decay rate of SWCNTs coupled to plasmonic nanocavities
γ_{R}	radiative decay rate of reference SWCNTs on planar gold mirror
γ_{NR}	non-radiative decay rate of reference SWCNTs on planar gold mirror
γ_{M}	metal loss rate
F_{p}	Purcell factor
η_{off}	quantum yield of reference SWCNTs
η_{on}	quantum yield of SWCNTs coupled to plasmonic nanocavities

Supplementary Table 1. **Definition of all parameters involved in the derivation of Purcell factor and quantum yield of SWCNT coupled to plasmonic nanocavities.**

Supplementary Note 4. External collection efficiency

We measured the external collection efficiency of our setups excluding the collection efficiency into the first lens that was already described in **Supplementary Fig. 2** above. For recording spectra and determining the EF factors we have used the spectrometer with attached CCD camera. For recording time-resolved emission rates or antibunching signatures we have sent the light directly into avalanche photodiode (APD) detectors without using the spectrometer system. All the values are calibrated by 860 nm laser beam sent to each component. As listed in **Supplementary Table 2**, we estimate the external collection efficiency for both APD and CCD with spectrometer configurations.

	**APD**	**CCD with spectrometer**
NIR objective transmission	82%	82%
Cryostat optics	50%	50%
MM fiber coupling	80%	80%
10 nm bandpass filter	45%	45%
APD quantum efficiency	47%	
MM fiber in-coupling		90%
300 gr/mm grating		37%
Aluminum coated mirrors × 3		$(71\%)^3 = 35\%$
CCD camera quantum efficiency		45%
**Total collection efficiency**	7 ± 2 %	0.77 ± 0.2 %

Supplementary Table 2. **Transmission/reflection losses or yield of optical components.** All values are extracted from experimental measurements using 860 nm laser light except the avalanche photodiode and CCD detector efficiencies which are taken from the manufacturer datasheet. Note that missing entries mean that the specific component is not part of the optical pathway.

Supplementary Note 5. Optical properties of reference SWCNTs

To carry out quantitative spectroscopy on individual SWCNTs it is important to have a stable reference to calibrate plasmonic enhancement for coupled SWCNTs. It is well known that different SWCNTs can have optical properties that vary quite drastically from tube to tube even if they have the same chirality or are suspended in air[8]. In order to reduce any environmental interaction affecting the exciton photophysics we have implemented a dual strategy. On the one hand we have recently shown that

the exciton ZPL of PFO-BPy-wrapped SWCNTs is decoupled from 1D-phonon dephasing and follows the acoustic phonon localization induced by the helically-wrapped PFO-BPy, resulting in stable and narrow PL emission[9]. In addition, we have recently shown that high-quality Al_2O_3 grown by atomic layer deposition (ALD) can drastically reduce spectral diffusion effects due to interactions with the dielectric environment [10]. As a result, our reference SWCNTs have exceptionally stable optical properties that vary by less than 20% from tube to tube. **Supplementary Fig. 4** shows a statistical analysis of 20 reference SWCNTs that were located on the planar gold mirror with 60 nm SiO_2 and 2 nm Al_2O_3. **Supplementary Fig. 4a** shows the ZPL peak intensity of each SWCNT recorded at fixed excitation power of 0.6 mW. The reference SWCNTs show a narrow intensity distribution with an average value of 920 cts s^{-1} and a standard deviation (SD) of 170 cts s^{-1}, corresponding to 20.7% variation. **Supplementary Fig. 4b** shows that the T_1 lifetimes measured for 10 reference SWCNTs are distributed rather narrowly between 194-248 ps with an average value of τ_{off}=215 ps and an SD of 20 ps, corresponding to 9.3% variation. Likewise, **Supplementary Fig. 4d** shows ZPL linewidth values for 10 reference SWCNTs recorded at fixed excitation power of 25 μW with an average FWHM of 80 μeV, SD = 8.6 μeV, corresponding to a 10.8% variation. The outstanding stability of these reference SWCNTs with optical properties that vary only between 10-20% from tube to tube justifies that our statistical analysis determines meaningful values for the nanocavity enhanced properties of individual SWCNTs. This is particularly true since EF, *F*p and quantum yield values are plasmonically enhanced by more than one order of magnitude for each and every SWCNT, that is significantly larger than the variation in optical properties of the reference SWCNTs.

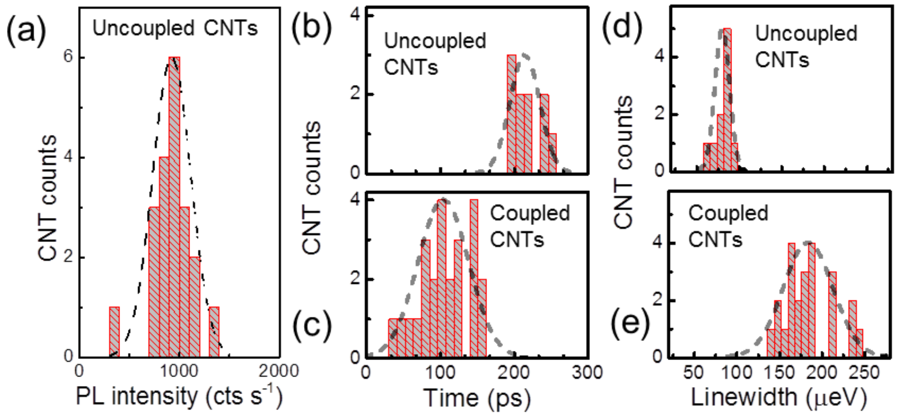

Supplementary Figure 4 Statistical analysis of reference SWCNTs located on the planar metal mirror with protecting dielectric. a, Histogram of time integrated PL intensity of 20 SWCNTs recorded at 0.6 mW. **b** and **c** Histograms of T_1 exciton recombination time for 10 reference SWCNTs (b) and 21 coupled SWCNTs (c). **d and e,** Full-width half-maximum (FWHM) of exciton ZPL for 10 reference (d) and 21 coupled SWCNTs (e).

Supplementary Note 6. Statistics of lifetime measurements

The TCSPC measurements have been carried out with a supercontinuum laser, coincidence electrons, and high timing resolution APDs as described in the methods section. **Supplementary Fig. 5** shows several of the time traces that have been recorded to create the statistical data presented in **Supplementary Fig. 4.** Corresponding deconvolution fits including the system response function are shown exemplarily in **Fig. 4g** of the main text.

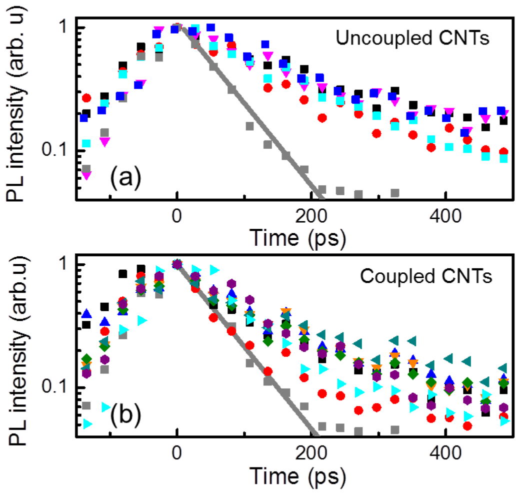

Supplementary Figure 5 Statistical analysis of coupled and reference carbon nanotubes. a and **b**, time-correlated single photon counting (TCSPC) measurement of reference (a) and coupled (b) SWCNTs, respectively. Grey data points and grey solid line represents the measured and fitted system response.

Supplementary Note 7. Zero-phonon-linewidth deconvolution procedure

To precisely determine the zero phonon line width of the E_{11} exciton emission we first note that it is largely unaffected by asymmetric phonon coupling for the case studied here of co-polymer wrapped SWCNTs, as is evident from the lineshape fits to the full theory shown above. We thus can assume that the FWHM can be determined from a single Lorentzian line fit, in particular at low optical pump powers. We have first measured the system response of the spectrometer (1800 groove/mm grating, focal length 0.75 m, slit width setting 20 μm) by sending laser light that is back-reflected from the sample surface through the same optical path as the E_{11} emission is sent. A single Gaussian fit to the system response yields FWHM = 25.4 μeV at 780 nm (**Supplementary Fig. 6a**), with R^2=0.996. This corresponds to a value of 23.4 μeV

at 890 nm where the E_{11} emission was recorded. Using mathematical deconvolution with a Voigt function one can largely eliminate the system response and can determine spectral width down to much lower linewidth, in particular at good signal to noise ratio. The Voigt deconvolution procedure is given by：

$$y = y_0 + A\frac{2\ln 2}{\pi^{3/2}}\frac{\omega_0}{\omega_{\mathrm{IRS}}}\int_{-\infty}^{\infty}\frac{\mathrm{e}^{-t^2}}{(\sqrt{\ln 2}\frac{\omega_0}{\omega_{\mathrm{IRS}}})^2+(2\sqrt{\ln 2}\frac{x-x_c}{\omega_0}-t)^2}\mathrm{d}t, \quad (17)$$

where y_0, ω_0, ω_{IRS}, x_c are background counts, FWHM of the emitter spectrum, FWHM of the instrument response, and the resonance frequency, respectively. **Supplementary Fig. 6 b-d** shows three examples of the deconvolution analysis for lowest pump powers in the experiment, with a minimum FWHM for the E_{11} emission of only 18 ± 3 μeV. Regarding the quality of this deconvolution procedure to eliminate the spectrometer response function we like to point out that the signal to noise ratio (SNR) reaches up to 14 (12 dB) in **Supplementary Fig. 6d** and 5 (7 dB) in **Supplementary Fig. 6b**. It was shown by Blass[11] that at SNR=100 deconvolved values down to 2-2.5 times narrower than the spectrometer resolution can be reliably extracted. Likewise, linewidth values up to 1.5 times (33%) narrower than the spectral resolution limit require SNR values of about 5-10. The extracted FWHM values of 18-21 μeV for the three lowest pump powers are only 10%-14% below the resolution limit. At the same time the corresponding SNR values vary between 5 and 14 and are thus large enough to produce reliable results from the Voigt deconvolution. We note that larger FWHM above 100 μeV are no longer significantly influenced by the system response function and single Lorentzian or Voigt function analysis results in the same estimate for the FWHM.

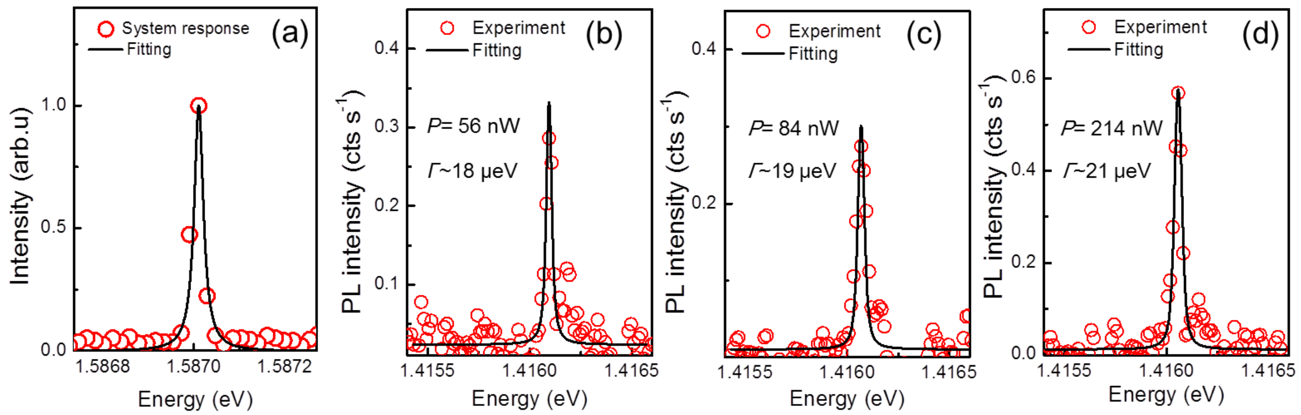

Supplementary Figure 6 Deconvolution procedure to determine the FWHM of the ZPL. a, System response function for 780 nm laser light that fits with a single Gaussian. The E_{11} exciton emission (red circles) was taken at excitation pump powers of 56 nW (b) 84 nW (c) and 214 nW (d). The black solid lines are fit to the Voigt function defined in Supplementary Equation 6 that includes the Lorentzian shown in (a). All data are recorded at 3.8 K.

Supplementary Note 8. Modelling exciton emission spectra in presence of acoustic phonon confinement

As we have recently shown, SWCNTs can display narrow and symmetric ZPL with well-resolved acoustic-phonon sidebands at low temperatures when they are wrapped with a polyfluorene co-polymer[9]. The corresponding phonon sideband-resolved emission spectra as well as their characteristic temperature dependence following the phonon occupation function can be fully described by a non-ohmic model that in addition to exciton localization assumes extrinsic acoustic-phonon localization at the nanoscale (5-10 nm length, 1-2 meV acoustic barrier heights), that is mediated by the copolymer backbone. Technically, the peculiar lineshapes of such quantum dot like excitons can be calculated by taking the mirror image of the absorption spectrum, which itself is derived from the imaginary part of the Fourier transform of the linear susceptibility $\chi(t)$ in response to a δ-shaped laser pulse, given by:

$$\chi(\mathrm{t}) = -i\mathrm{e}^{-i\bar{\Omega}t}\chi_T(t)\chi_0(t) \tag{18}$$

where $\bar{\Omega} = \Omega - \sum_q |\gamma_{\mathrm{s}}(q)|^2 \omega_{\mathrm{s}}(q)$ is the polaron-corrected transition frequency with Ω denoting the bare energy of the quantum dot state[12,13]. The temperature-dependent $\chi_T(t)$ and temperature-independent $\chi_0(t)$ functions in **Supplementary Equation 11** can be expressed as

$$\chi_{\mathrm{T}}(t) \propto i\exp(\textstyle\sum_{\mathrm{q}} |\gamma_{\mathrm{s}}(q)|^2 \left[-n_{\mathrm{s}}(q)\left|e^{-i\omega_{\mathrm{s}}(q)t} - 1\right|^2\right]) \tag{19}$$

$$\chi_0(t) \propto i\exp(\textstyle\sum_q |\gamma_{\mathrm{s}}(q)|^2 \left[e^{-i\omega_{\mathrm{s}}(q)t} - 1\right]) \tag{20}$$

where $n_{\mathrm{s}}(q) = [\exp(\frac{\hbar\omega_{\mathrm{s}}(q)}{k_{\mathrm{B}}T}) - 1]^2$ is the phonon occupation number, $\gamma_S(q) = g_S(q)/\omega_{\mathrm{s}}(q)$ with $\omega_{\mathrm{s}}(q)$ describing the dispersion relation of the stretching mode. The exciton-phonon coupling matrix element $g_S(q)$ is given by the product of the deformation potential coupling $G(q)$ of the stretching mode and the acoustic phonon confinement modified exciton form factor

$$F(\omega_{\mathrm{i}}) = D\sqrt{L}\nu/\omega_{\mathrm{i}} \int \mathrm{d}z |\psi(z)|^2 W_i(z) \tag{21}$$

Where $\psi(z)$ describes the envelope wave function of localized excitons, $W_i(z)$ describes the orthonormal acoustic mode wave functions, and L the nanotube length[9,14]. This model can fully describe the ZPL and its phonon wings as well as their temperature dependence, as shown in **Supplementary Fig. 7**. We note that the lower-energy wing in each spectrum corresponds to emission of a blue detuned photon due to phonon absorption and scales with the phonon occupation number $n_{\mathrm{s}}(q)$, whereas the higher-energy wing corresponds to emission of red detuned photons due to phonon emission and its probability scales with

$n_s(q)+1$. Thus at lowest temperatures predominantly the Stokes component is visible (see **Fig. 5c** for an example at 20 K) while the anti-Stokes component increases drastically with increasing temperature as shown in **Supplementary Fig. 7**. One can further obtain the relative height of the two side peaks from the relation:

$$\frac{I_{\mathrm{L}}}{I_{\mathrm{H}}} = \frac{n_{\mathrm{s}}}{n_{\mathrm{s}}+1}\exp\left(\frac{-\varepsilon}{k_{\mathrm{B}}T_{\mathrm{eff}}}\right), \tag{22}$$

where I_L, I_H, ε, k_B and T_{eff} are the PL intensity of lower-energy wing and higher-energy wing, and the energy splitting between the ZPL and each side wing, the Boltzmann factor and effective temperature, respectively. We found that the relative height of the lower-energy and higher-energy shifted acoustic phonon wings follows, as expected, a Boltzmann activation energy which is nearly equal to an energy splitting of about 1.8 meV between the peaks that corresponds to the acoustic phonon barrier height. The outcome of this lineshape analysis is a precise calibration of the local temperature seen by an individual carbon nanotube and induced by the plasmonic nanocavity mode. The temperature read out via the bosonic nature of the acoustic phonon population can thus precisely reflect the local heating induced by an individual plasmonic nanostructure. We note that this interplay between excitons, plasmons, and phonons can be well described in the temperature range from 4K to about 130-150K with the non-Ohmic model, above which the broad lineshape of the SWCNT does not show any longer a change with temperature since the acoustic phonon confinement is fully thermalized.

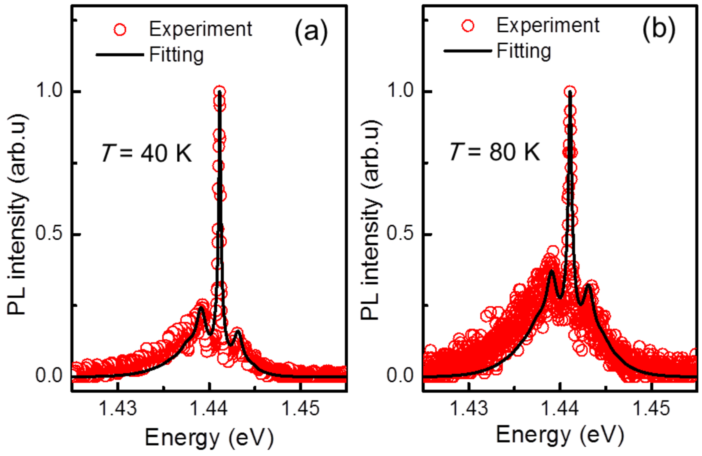

Supplementary Figure 7. Lineshape analysis of the exciton emission under acoustic phonon confinement of co-polymer wrapped SWCNTs. The PL spectra (red circles) have been measured at a sample temperature of 40 K (a) and 80K (b), respectively, while the pump power was kept moderately low. The solid lines are fits to the theory described in the text where the relative strength of the Stokes and anti-Stokes phonon wings is determined by the temperature parameter in the phonon occupation factor.

Supplementary References

1. Kinkhabwala, A. *et al.* Large single-molecule fluorescence enhancements produced by a bowtie nanoantenna. *Nat. Photonics* **3**, 654–657 (2009).
2. Johnson, P. B. & Christy, R.-W. Optical constants of the noble metals. *Phys. Rev. B* **6**, 4370 (1972).
3. Palik, E. D. *Handbook of optical constants of solids*. **3**, (Academic press: 1998).
4. Koenderink, A. F. On the use of Purcell factors for plasmon antennas. *Opt. Lett.* **35**, 4208–4210 (2010).
5. Russell, K. J., Liu, T.-L., Cui, S. & Hu, E. L. Large spontaneous emission enhancement in plasmonic nanocavities. *Nat. Photonics* **6**, 459–462 (2012).
6. Hofmann, M. S. *et al.* Bright, long-lived and coherent excitons in carbon nanotube quantum dots. *Nat. Nanotechnol.* **8**, 502–505 (2013).
7. Lakowicz, J. R. *Principles of fluorescence spectroscopy*. (Springer Science & Business Media: 2013).
8. Sarpkaya, I. *et al.* Prolonged spontaneous emission and dephasing of localized excitons in air-bridged carbon nanotubes. *Nat. Commun.* **4**, 2152 (2013).
9. Sarpkaya, I. *et al.* Strong Acoustic Phonon Localization in Copolymer-Wrapped Carbon Nanotubes. *ACS Nano* **9**, 6383–6393 (2015).
10. Li, X. *et al.* Nonmagnetic Quantum Emitters in Boron Nitride with Ultranarrow and Sideband-Free Emission Spectra. *ACS Nano* **11**, 6652–6660 (2017).
11. Blass, W. *Deconvolution of absorption spectra*. (Elsevier: 2012).
12. Krummheuer, B., Axt, V. M. & Kuhn, T. Theory of pure dephasing and the resulting absorption line shape in semiconductor quantum dots. *Phys. Rev. B* **65**, 195313 (2002).
13. Vagov, A., Axt, V. & Kuhn, T. Electron-phonon dynamics in optically excited quantum dots: Exact solution for multiple ultrashort laser pulses. *Phys. Rev. B* **66**, 165312 (2002).
14. Vialla, F. *et al.* Unifying the low-temperature photoluminescence spectra of carbon nanotubes: The role of acoustic phonon confinement. *Phys. Rev. Lett.* **113**, 057402 (2014).
